# Racial Differences in Non-variceal Upper Gastrointestinal (GI) Bleeding: A Nationwide Study

**DOI:** 10.7759/cureus.61982

**Published:** 2024-06-09

**Authors:** Raissa Nana Sede Mbakop, Arnold N Forlemu, Chidiebube Ugwu, Elizabeth Soladoye, Kikelomo Olaosebikan, Emeka S Obi, Dominic Amakye

**Affiliations:** 1 Internal Medicine, Piedmont Athens Regional Medical Center, Athens, USA; 2 Gastroenterology and Hepatology, The Brooklyn Hospital Center, Brooklyn, USA; 3 Public Health, East Tennessee State University, Johnson City, USA

**Keywords:** usa: united states of america, usa, non-variceal upper gastrointestinal bleeding, nationwide study, upper gi bleed, racial differences

## Abstract

Background and aims

Knowledge about the impact of race on non-variceal upper GI bleeding (NVUGIB) is limited. This study explored the racial differences in the etiology and outcome of NVUGIB.

Methods

We conducted a study from 2009 to 2014 using the Nationwide Inpatient Sample (NIS) database. NIS is the largest publicly available all-payer inpatient database in the USA with more than seven million hospital stays each year. The International Classification of Diseases, Ninth Revision, Clinical Modification (ICD-9-CM) codes for NVUGIB, esophagogastroduodenoscopy (EGD) and demographics were obtained. The outcomes of interest were in-hospital mortality, hospital length of stay (HLOS), total hospital charges, admission to the intensive care unit (ICU), and patient disposition. Analysis was conducted using Chi-square tests and Tukey multiple comparisons between groups.

Results

Among 1,082,516 patients with NVUGIB, African American and Native Americans had the highest proportions of hemorrhagic gastritis/duodenitis (8.2% and 4.2%, respectively) and Mallory-Weiss bleeding (10.4% and 5.4%, respectively; p<0.01). African Americans were less likely to get an EGD done within 24 hours of admission compared to Whites and Latinxs (45.9% vs 50.1% and 50.4%, respectively; p<0.001). In-hospital mortality was similar among African Americans, Latinxs, and Whites (5.8% vs 5.6% vs 5.9%, respectively; p=0.175). Asian/Pacific Islanders and African Americans were more likely to be admitted to the ICU (9.6% and 9.0%, respectively; p<0.001). Moreover, African Americans had a longer HLOS compared to Latinxs and Whites (7.5 vs 6.5 and 6.4 days, respectively; p<0.001). Conversely, Asian/Pacific Islanders and Latinx incurred the highest hospital total charges compared to African Americans and Whites ($81,821 and $69,267 vs $61,484 and $53,767, respectively; p<0.001).

Conclusion

African Americans are less likely to receive EGD within 24 hours of admission and are more likely to be admitted to the ICU with prolonged hospital lengths of stay. Latinxs are more likely to be uninsured and incur the highest hospital costs.

## Introduction

Gastrointestinal (GI) bleeding is the most common GI diagnosis requiring admission. It accounts for over half a million admissions each year with $5 billion of direct costs [1]. Roughly half of GI bleeds are upper GI bleeding. Upper GI bleeding refers to bleeding from a GI source proximal to the ligament of Treitz [2]. Its incidence is estimated at 80-150 per 100,000 people. Despite a noted decrease in its incidence in recent years, it remains a serious disease with an estimated mortality of 5% [3]. Race has been shown to have an impact on the clinical presentation of many diseases, access to certain procedures, and outcomes of these diseases. For example, studies have shown that African Americans have worse outcomes after trauma [4], Asians represent the minority with the most admissions for acute cholangitis [5], and Latinxs and Native Americans have lower odds of undergoing esophagogastroduodenoscopy (EGD) for upper GI bleeding in rural hospitals [6]. Despite an increasing interest in racial differences in upper GI bleeding [7-9], the literature is limited on how race affects the etiologies and outcomes of non-variceal upper GI bleeding (NVUGIB). Our study aimed to elucidate differences in the etiologies of non-variceal upper GI bleeding among various racial groups and determine if there are any differences in outcomes such as in-hospital mortality, hospital length of stay (HLOS), admission to the intensive care unit (ICU), and total hospital charges.

This article was previously presented as a meeting abstract at the 2021 Digestive Disease Week (DDW) on May 21, 2021.

## Materials and methods

We used data from the Nationwide Inpatient Sample (NIS) database covering the period from 2009 to 2014 to conduct our study. NIS is the largest publicly available all‐payer inpatient database in the USA, with more than seven million hospital stays each year, as a part of the Healthcare Cost and Utilization Project (HCUP). It is designed to be nationally representative, with a 20% stratified sample of hospitalizations obtained from 46 states, comprising more than 97% of the US population, and it captures a significant number of community hospitals in the USA. The HCUP database contains de-identified data on nationwide hospital admissions including demographics, clinical data, comorbidities, discharge diagnoses, procedures, outcomes, and hospitalization costs. The NIS database lists patients with a primary discharge diagnosis up to 29 secondary discharge diagnoses and 15 procedure codes. International Classification of Diseases, Ninth Revision, Clinical Modification (ICD-9-CM) codes for NVUGIB and EGD were obtained (Table 1). Similarly, information on age, sex, race, insurance type, and chronic medical conditions was also obtained. Participants included in this study were aged 18 and above. Patients with a history of liver cirrhosis and patients admitted for anything other than NVUGIB were excluded from this study.

**Table 1:** ICD-9-CM diagnostic and procedure codes

Description	ICD-9-CM code
Bleeding esophageal ulcer	530.21
Mallory Weiss	530.7
Bleeding peptic ulcer (gastric or duodenal)	531.0x, 531.2x, 531.4x, 531.6x, 532.0x, 532.2x, 532.4x, 532.6x, 533.0x, 533.2x, 533.4x, 533.6x, 534.0x, 534.2x, 534.4x, 534.6x
Gastritis/duodenitis with hemorrhage	535.01, 535.11, 535.21, 535.31, 535.41, 535.51, 535.61, 535.71
Angiodysplasia (stomach or duodenum) with hemorrhage	537.83, 569.85
Dieulafoy’s lesion of the stomach or duodenum	569.86
Esophagogastroduodenoscopy	45.13, 45.14, 45.16, 42.23, 44.13, 42.33, 44.43
Mechanical ventilation	967.0, 967.1, 967.2
Vasopressor infusion	00.17

The outcomes we evaluated were in-hospital mortality, HLOS, total hospital charges, admission to the intensive care unit (ICU), and patient disposition. Statistical analysis was conducted using Statistical Product and Service Solutions (SPSS) software (version 24.0; IBM SPSS Statistics for Windows, Armonk, NY). Chi-square tests and analysis of variance techniques were used to analyze categorical proportions and continuous variable means, respectively. The Tukey test was used to perform pairwise comparisons between multiple groups. Statistical significance was set at a two-tailed p<0.05 for all tests. Estimates were presented as odds ratios and 95% confidence intervals.

## Results

Among the 1,082,516 patients with NVUGIB, 145,949 (14.7%) were African Americans, 95,736 (9.6%) were Latinxs, 692,348 (69.6%) were Whites, and 60,633 (6.1%) were from other racial groups. The prevalence of NVUGIB among African Americans significantly increased from 14.1% in 2009 to 17.2% in 2014 (p<0.001), while that of the other races decreased from 17.1% to 16.5% during the same period. African Americans and Native Americans had the highest proportions of hemorrhagic gastritis/duodenitis (8.2% and 4.2%, respectively) and Mallory-Weiss bleeding (10.4% and 5.4%, respectively; p<0.01; Table 2).

**Table 2:** Racial differences among etiologies and outcomes of non-variceal upper GI bleeding GI, Gastrointestinal

Parameter	African American	Latinx	White	Asian/Pacific Islander	Native American	Other races	P value
Bleeding etiology peptic ulcer	113,889 (78.0)	65,924 (68.9)	534,406 (77.2)	20,560 (77.4)	5,218 (73.7)	19,950 (73.9)	<0.01
Gastritis/duodenitis	12,009 (8.2)	7,624 (8.0)	51,635 (7.5)	1,929 (7.3)	737 (10.4)	1,880 (7.0)	0.01
Mallory-Weiss	6,086 (4.2)	3,910 (4.1)	22,744 (3.3)	771 (2.9)	381 (5.4)	993 (3.7)	0.005
Esophageal ulcer	1,740 (1.2)	1,480 (1.5)	11,881 (1.7)	336 (1.3)	109 (1.5)	355 (1.3)	<0.01
Dieulafoy lesion	163 (0.1)	75 (0.1)	840 (0.1)	43 (0.2)	9 (0.1)	28 (0.1)	0.002
Angiodysplasia	5,229 (3.6)	2,452 (2.6)	23,291 (3.4)	409 (1.5)	168 (2.4)	654 (2.4)	<0.01
Bleeding patient characteristics age (mean in years)	61.6	58.3	67.9	66.7	57.8	62.0	0.019
Insurance type							<0.001
Medicare	80,199 (56.7)	40,949 (44.6)	449,973 (66.7)	14,007 (54.2)	3,058 (48.5)	13,136 (50.3)	
Medicaid	25,250 (17.9)	22,568 (24.6)	51,320 (11.3)	3,990 (15.4)	1,510 (24.0)	4,668 (17.9)	
Private insurance	24,492 (17.3)	17,201 (18.7)	139,043 (20.6)	6,491 (25.1)	1,165 (18.5)	5,764 (22.1)	
Self-pay	10,365 (7.3)	9,979 (10.9)	31,430 (4.7)	1,256 (4.9)	520 (8.3)	2,327 (8.9)	
Other insurance	1,050 (0.7)	1,170 (1.3)	3,042 (0.5)	93 (0.4)	48 (0.8)	244 (0.9)	
Sex							0.001
Male	72,410 (49.6)	49,230 (51.4)	341,306 (49.3)	13,928 (52.4)	3,671 (51.8)	14,213 (52.7)	
Female	73,522 (50.4)	46,503 (48.6)	350,976 (50.7)	12,641 (47.6)	3,411 (48.2)	12,768 (47.3)	
Obesity	14,972 (10.3)	9,135 (9.5)	64,315 (9.3)	1,066 (4.0)	750 (10.6)	2,217 (8.2)	<0.001
EGD within 24 hours	34,147 (45.9)	23,900 (50.1)	179,436 (50.4)	7,906 (55.0)	1,774 (49.6)	6,510 (49.5)	0.001
Outcomes, inpatient dead	8,471 (5.8)	5,346 (5.6)	40,754 (5.9)	1,918 (7.2)	424 (6.0)	1,855 (6.9)	0.175
Hospital LOS (days)	7.5	6.5	6.4	7.2	5.9	7.5	<0.001
Hospital charges ($)	61,484	69,267	53,767	81,821	47,985	68,709	<0.001
ICU admission	13,136 (9.0)	7,637 (8.0)	52,801 (7.6)	2,540 (9.6)	617 (8.7)	2,803 (10.4)	0.001
Disposition							0.001
Home	83,004 (60.4)	64,234 (71.2)	381,647 (58.7)	16,179 (65.8)	4,698 (70.7)	15,940 (63.5)	
Home health or SNF	51,171 (37.3)	24,141 (26.8)	260,515 (40.0)	8,181 (33.3)	1,774 (26.7)	8,652 (34.5)	
Left against medical advice	3,142 (2.3)	1,820 (2.0)	8,483 (1.3)	224 (0.9)	174 (2.6)	507 (2.0)	

African Americans were less likely to get an EGD done within 24 hours of admission compared to Whites and Latinxs (45.9% vs 50.1% and 50.4%, respectively; p<0.001). In-hospital mortality was similar among African Americans, Latinxs, and Whites (5.8% vs 5.6% vs 5.9%, respectively; p=0.175). Asian/Pacific Islanders and African Americans were more likely to be admitted to the ICU (9.6% and 9.0%, respectively; p<0.001). African Americans and Whites were more likely to be discharged to a short or long-term rehabilitation facility compared to Latinxs (37.3% and 40.0% vs 26.8%, p<0.001). Moreover, African Americans had a longer HLOS compared to Latinxs and Whites (7.5 vs 6.5 and 6.4 days, respectively; p<0.001,). Conversely, Asian/Pacific Islanders and Latinxs incurred the highest hospital total charges compared to African Americans and Whites ($81,821 and $69,267 vs $61,484 and $53,767, respectively; p<0.001).

## Discussion

Using a large nationwide data source, this study strongly suggests that race is a significant determinant of the incidence, etiology, and outcome of NVUGIB. Upper GI bleeding is responsible for over 200,000 hospital admissions annually in the United States [1]. Multiple studies have shown that peptic ulcer disease is the most common cause of NVUGIB, accounting for about 31-67% of cases [3], followed by erosive disease, esophagitis, malignancy, and Mallory-Weiss tears. Uncommon causes of NVUGIB such as Dieulafoy lesion, hemobilia, angiodysplasia, vascular-enteric fistula, and gastric antral vascular ectasia account for 2% to 8% of cases. Little is known about the racial differences in the frequency of various etiologies of NVUGIB. In our study, African Americans and Asians/Pacific Islanders had the highest rates of NVUGIB secondary to peptic ulcer disease when compared to other races (78% and 77.4%, respectively; p<0.01). This can be explained partly by the known higher rates of Helicobacter pylori (Hp) infection in African Americans [10]. Conversely, Asians/Pacific Islanders are not known to have high rates of Hp infection. However, together with African Americans, they are more susceptible to Hp and, therefore, have the highest rates of Hp-associated peptic ulcer disease when compared to other races [11]. One of the reasons suggested for African Americans being more susceptible to Hp is that they are three times more likely to have the Cag-A genotype of Hp, which is the most virulent form of Hp [11]. Similarly, gastritis and duodenitis were the most frequent (10.4% and 8.2% vs 8.0%, 7.5%, 7.3%, and 7.0%; p=0.01) in Native Americans and African Americans; this appears to correspond to the higher rates of Hp infection. Endoscopy plays a pivotal role in the diagnosis and management of NVUGIB such that the American College of Gastroenterology recommends that an endoscopy be performed within 24 hours of admission [12]. In effect, endoscopy affords the possibility of various therapeutic modalities including clips, argon plasma coagulation, electrocoagulation, and hemostatic spray. When these procedures are done within 24 hours of admission, it results in reduced mortality [13] and a lower need for surgery [14,15]. However, in our study, we noted that African Americans were less likely to get an EGD done within 24 hours of admission when compared to Whites and Latinxs (45.9% vs 50.1% and 50.4%, respectively; p<0.001). In 2011, Wysocki et al. in their nationwide analysis of risk factors for mortality in upper GI bleeding, had similar findings [13]. They noted that African Americans were less likely to receive an EGD within one day of admission. The exact reason for this is uncertain. Some have postulated that this may be secondary to the language barrier [16], behavioral patterns [6], or even the implicit bias of providers [17]. To ascertain this, specific studies will need to be done in the future. Despite the differences in access to early EGD in our study, we did not find any difference in mortality among the various races. This suggests that the timeliness of EGD did not have a major impact on this large study sample. The HLOS of African Americans was, however, longer, which could be attributed to the delay in getting an EGD. Asian/Pacific Islanders and Latinxs incurred the highest hospital costs in our study. Wollenman et al., in their study on the impact of ethnicity on upper GI bleeding, found that Latinxs tended to have a higher rebleeding rate [17]. This could partly account for their higher hospital costs. In the era of massive interest and research in artificial intelligence that relies heavily on algorithms, race must be considered when attempting to predict the etiologies and outcomes of NVUGIB. Our study had a few limitations. The NIS data do not contain information on disease duration, disease severity including potential contraindications to EGD (e.g., severe pulmonary compromise, severe coagulopathy), as well as medications used by patients. Hence, their associations with the etiologies of NVUGIB and impact on outcomes of interest could not be ascertained. Similarly, incorrect ICD coding could lead to erroneous inclusion of patients with variceal bleeding. However, the codes used are validated, and, with the large dataset used, this is unlikely to significantly affect the overall result.

## Conclusions

By examining a nationwide diverse population, we conclude that NVUGIB does follow certain trends. The prevalence of NVUGIB in African Americans has been rising, and they are less likely to receive EGD within 24 hours of admission. African Americans are also more likely to be admitted to the ICU with prolonged hospital lengths of stay. Latinxs are more likely to be uninsured and incur higher hospital costs. While race appears to play a key role in the presentation, management, and outcomes of NVUGIB, it is less likely to influence overall mortality. There are likely other factors that influence the overall outcome and further studies are required to explore these factors.
